# Fast Neutron-Induced Enhancement of the I8 Exciton Emission in ZnO Bulk Single Crystals

**DOI:** 10.3390/nano16160978

**Published:** 2026-08-09

**Authors:** Mohammad M. Zeidan, Sufian Abedrabbo

**Affiliations:** 1 Engineering Department, Higher Colleges of Technology, Abu Dhabi P.O. Box 25026, United Arab Emirates; 2 Department of Physics, Khalifa University, Abu Dhabi P.O. Box 127788, United Arab Emirates

**Keywords:** zinc oxide, neutron, transmutation, gallium, photoluminescence, Zn-Polar Face

## Abstract

Zinc oxide (ZnO) is a wide-bandgap semiconductor with important optoelectronic, photonic, and radiation-related applications. In this work, hydrothermally grown ZnO bulk single crystals were irradiated with fast neutrons and characterized using low-temperature photoluminescence (PL) spectroscopy to investigate irradiation-induced changes in the Ga-related donor-bound exciton (I_8_). The Zn-polar surfaces were exposed to fast neutrons for irradiation durations of 2 and 5 min. The PL measurements showed a reproducible enhancement of the Ga-related I_8_ emission intensity, with increases of approximately 33% after 2 min and 82% after 5 min relative to the unirradiated reference crystal. The selective enhancement of the I_8_ emission is consistent with increased Ga-related donor activity associated with the proposed Zn-to-Ga neutron transmutation mechanism, although irradiation-induced defect formation and redistribution may also contribute to the observed optical response. Under the irradiation conditions investigated, fast-neutron exposure produced substantial enhancement of the I_8_ emission within only a few minutes. However, direct quantitative comparison with previously reported slow-neutron irradiation should be interpreted with caution because the two studies employed different neutron energies, fluences, and irradiation conditions. These findings demonstrate the potential of fast-neutron irradiation for modifying the low-temperature optical response of hydrothermally grown ZnO and provide a foundation for future investigations of neutron-induced defect and donor engineering in wide-bandgap oxide semiconductors.

## 1. Introduction

Zinc oxide (ZnO) is a wide-bandgap semiconductor that has attracted significant attention because of its distinctive optical, electronic, and piezoelectric properties. ZnO crystallizes in the hexagonal wurtzite structure and possesses a direct bandgap of approximately 3.37 eV together with a large exciton binding energy, making it highly suitable for optoelectronic and photonic applications [1,2,3,4,5,6,7,8,9,10,11,12,13,14,15,16]. In addition to its optical transparency and strong near-band-edge emission, ZnO exhibits high chemical stability, excellent radiation hardness, and favorable thermal properties, which further enhance its suitability for sensors, ultraviolet emitters, photovoltaics, transparent conductive films, and radiation-detection technologies [5,6,7,8,9,10,11,12,13]. Furthermore, the strong piezoelectric characteristics of ZnO have enabled its application in actuators, sensors, and energy-harvesting devices [17,18,19,20,21,22,23,24,25,26,27,28,29,30].

The photoluminescence (PL) properties of ZnO bulk single crystals have been extensively investigated because they provide valuable information regarding excitonic transitions, impurity incorporation, and defect-related recombination processes [5,10,11,12,23,31]. The PL spectrum of ZnO is typically dominated by near-band-edge (NBE) excitonic emissions together with defect-related luminescence features. Among these emissions, donor-bound excitonic transitions are particularly sensitive to impurity concentration and lattice disorder [10,11,12,23,31,32]. The gallium-related donor-bound exciton (I_8_) is commonly observed in hydrothermally grown ZnO crystals and serves as an important optical probe for investigating gallium-related donor activity and recombination mechanisms within the ZnO lattice [11].

**Figure 1:**
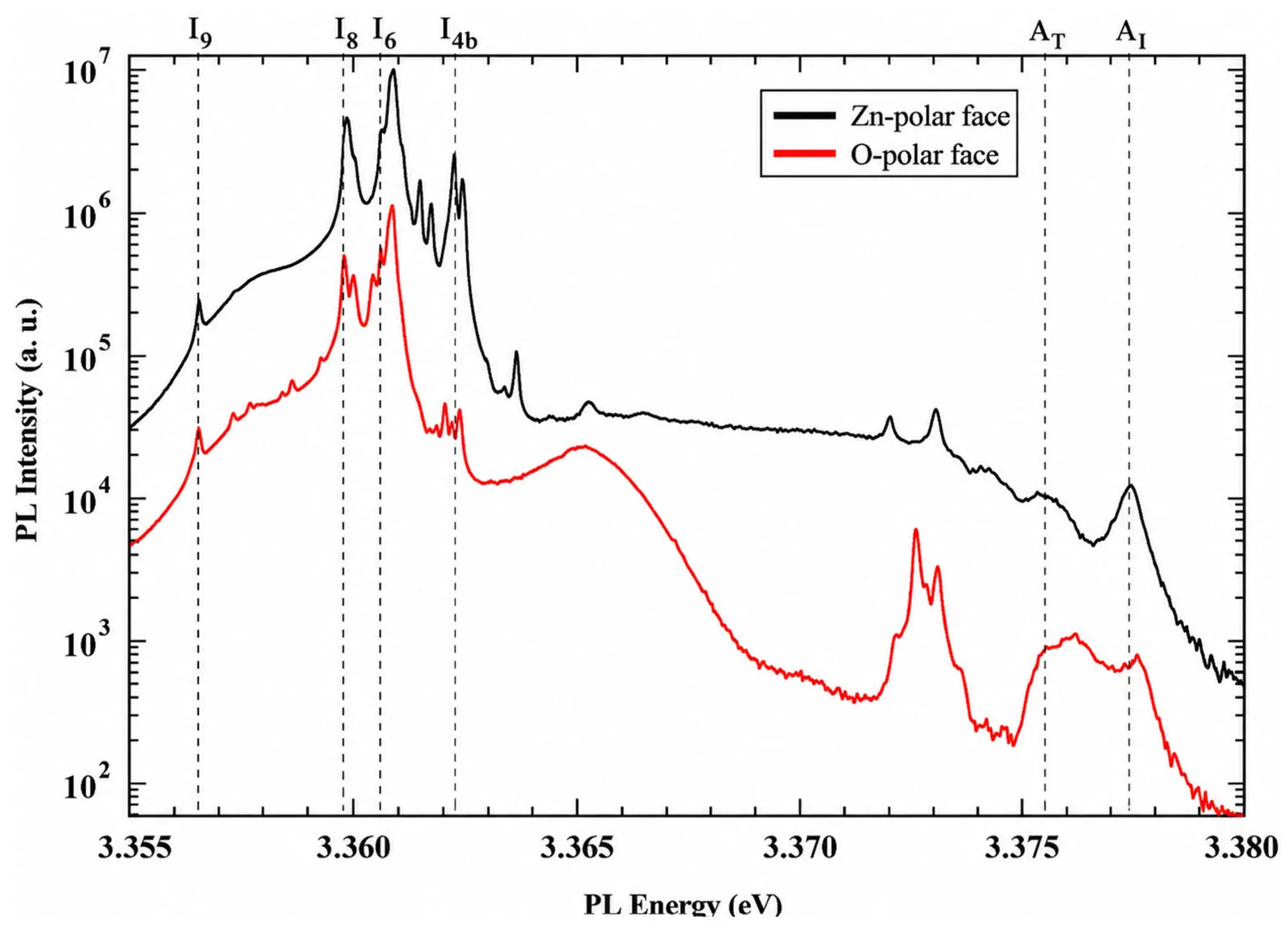
Low-temperature (4 K) photoluminescence (PL) spectra of hydrothermally grown ZnO bulk single crystals measured from the Zn-polar (0001) and O-polar (000$\bar{1}$) under 325 nm He–Cd laser excitation. The spectra illustrate the differences in near-band-edge excitonic emission behavior between the two polar crystal orientations.

ZnO exhibits two principal polar crystal orientations, namely the Zn-polar (0001) and O-polar (000$\bar{1}$) surfaces, which exhibit distinct optical and structural characteristics owing to differences in surface termination and defect distribution [11,12,23,31,32]. These polar surfaces exhibit different optical and structural characteristics due to variations in surface termination and defect distribution. In particular, the Zn-polar face generally exhibits stronger and sharper near-band-edge excitonic emission compared with the O-polar face [10,11,12,23,31,32,33]. Figure 1 illustrates the low-temperature PL spectra measured from both polar orientations, highlighting the stronger excitonic response of the Zn-polar surface investigated in the present work.

In addition to near-band-edge excitonic transitions, ZnO bulk crystals commonly exhibit broad deep-level emission (DLE) bands associated with intrinsic lattice defects such as oxygen vacancies, zinc interstitials, and antisite defects [31,32,33]. Since both excitonic and defect-related emissions are highly sensitive to crystal quality and lattice modification, PL spectroscopy provides an effective technique for evaluating irradiation-induced changes in ZnO optical behavior.

Radiation hardness is an important characteristic of ZnO for applications operating in high-radiation environments [10,11,12,23,33]. Nevertheless, exposure to ionizing radiation can introduce structural defects into the ZnO lattice, including vacancies, interstitials, dislocations, and defect complexes, which can significantly alter the optical and electrical properties of the material [23,31,32,33]. Previous investigations have demonstrated that neutron irradiation can modify the PL response of ZnO through lattice modification and transmutation-related processes [10]. In particular, neutron interactions with zinc isotopes can lead to the formation of gallium donor species, which influence donor-bound excitonic recombination and near-band-edge emission intensity.

In addition to neutron irradiation, energetic electrons, protons, gamma rays, and ion beams have also been employed to modify the structural and optical properties of ZnO. These studies demonstrate that irradiation-induced defect formation and defect evolution can significantly influence excitonic and defect-related luminescence, although the resulting optical response depends strongly on the radiation source, energy, fluence, and material characteristics [34,35,36,37]. This broader context provides an important framework for interpreting the fast-neutron irradiation effects investigated in the present work.

The present work investigates the enhancement of the gallium-related I_8_ donor-bound exciton in hydrothermally grown ZnO bulk single crystals using fast-neutron irradiation. Although neutron transmutation doping has previously been investigated in ZnO, relatively few studies have examined the influence of fast-neutron irradiation on the low-temperature excitonic photoluminescence of hydrothermally grown ZnO bulk single crystals. To the best of our knowledge, this is the first study to demonstrate a significant enhancement of the Ga-related I_8_ donor-bound exciton following short-duration (2–5 min) fast-neutron irradiation. The present work therefore provides new insight into the influence of fast neutrons on the optical properties of ZnO and enables comparison with previously reported slow-neutron irradiation studies under different irradiation conditions.

## 2. Experimental Procedure

### 2.1. Preparing Samples

High-purity hydrothermally grown ZnO bulk single-crystal wafers supplied by TEW Tokyo Denpa Co. Ltd. (TD), Morioka, Iwate, Japan, were used in this study. The wafers were sectioned into multiple specimens measuring approximately 3 × 3 mm^2^ with a thickness of 0.5 mm. All specimens were double-side polished prior to optical characterization [38]. ZnO crystallizes in the hexagonal wurtzite structure, consisting of alternating planes of zinc and oxygen atoms stacked along the crystallographic c-axis. These alternating atomic layers form two distinct polar surfaces when the crystal is terminated perpendicular to the c-axis: the Zn-polar (0001), terminated by zinc atoms, and the O-polar ($000\bar{1}$), terminated by oxygen atoms [31,32,33]. Previous investigations using X-ray photoemission spectroscopy and grazing-incidence surface X-ray diffraction have suggested that both polar surfaces are stabilized by approximately monolayer hydroxyl-group termination [39,40,41]. The presence of these hydroxyl groups is considered essential for stabilizing the polar surfaces, which would otherwise be electrostatically unstable [23,31,32,33].

To minimize surface contamination prior to irradiation and optical characterization, all samples underwent a standardized cleaning procedure. The cleaning process involved ultrasonic agitation sequentially in acetone, methanol, and isopropanol to remove residual organic contaminants, oils, and particulates from the sample surfaces. Following solvent cleaning, the samples were dried using high-purity nitrogen gas (N_2_) to eliminate residual solvent traces and prevent surface marking. This preparation procedure ensured clean and stable surfaces suitable for irradiation and subsequent photoluminescence measurements.

### 2.2. Photoluminescence Spectroscopy

Photoluminescence (PL) measurements were performed using a helium–cadmium (HeCd) laser (Kimmon Koha Co., Ltd., Tokyo, Japan) operating at a wavelength of 325 nm (3.81 eV) as the excitation source. The emitted PL signal from the ZnO crystals was analyzed using a Spex 1000M spectrometer equipped with a 1200 line/mm holographic diffraction grating for spectral dispersion of the emitted light. The dispersed signal was detected using a Hamamatsu photomultiplier tube (PMT) (Hamamatsu Photonics K.K., Shizuoka, Japan).

Low-temperature (4K) photoluminescence measurements were performed by mounting the samples inside a liquid-helium cryostat (Oxford Instruments plc, Abingdon, UK), enabling high-resolution observation of near-band-edge excitonic emission. The PL intensity was recorded using a dual-channel photon counter interfaced with LabVIEW software for signal acquisition and spectral analysis.

### 2.3. Irradiating Samples

Fast-neutron irradiation was carried out using the cyclotron facility at the Radiation Oncology Department, University of Washington (UW), USA. The fast neutrons were directed onto separate sets of pure ZnO bulk single crystals for irradiation durations of 2 and 5 min. The neutron beam possessed a field size of 10 × 10 cm^2^, an average neutron energy of 22.0 MeV, and a flux of approximately 1.4 × 10^8^ n/s.cm^2^. During irradiation, the distance between the neutron source and the samples was maintained at 150 cm.

The ZnO samples were positioned at the center of the neutron beam with the wafer surface oriented perpendicular to the crystallographic c-axis, ensuring direct irradiation of the Zn-polar (0001). This configuration enabled uniform neutron exposure across the irradiated surface and facilitated investigation of neutron-induced modifications to the optical properties of the Zn-polar orientation.

To evaluate the reproducibility of the irradiation-induced photoluminescence response, the experiments were repeated using multiple ZnO single-crystal samples prepared from the same hydrothermal wafer and irradiated under identical fast-neutron irradiation conditions. Reference photoluminescence spectra were likewise obtained from unirradiated samples originating from the same wafer to minimize sample-to-sample variations associated with crystal growth. All independently irradiated samples exhibited the same qualitative enhancement of the Ga-related I_8_ donor-bound exciton. The spectra presented in this work are representative of the reproducible optical response observed under the specified irradiation conditions.

## 3. Results

### 3.1. Photoluminescence Response Following Fast-Neutron Irradiation

**Figure 2:**
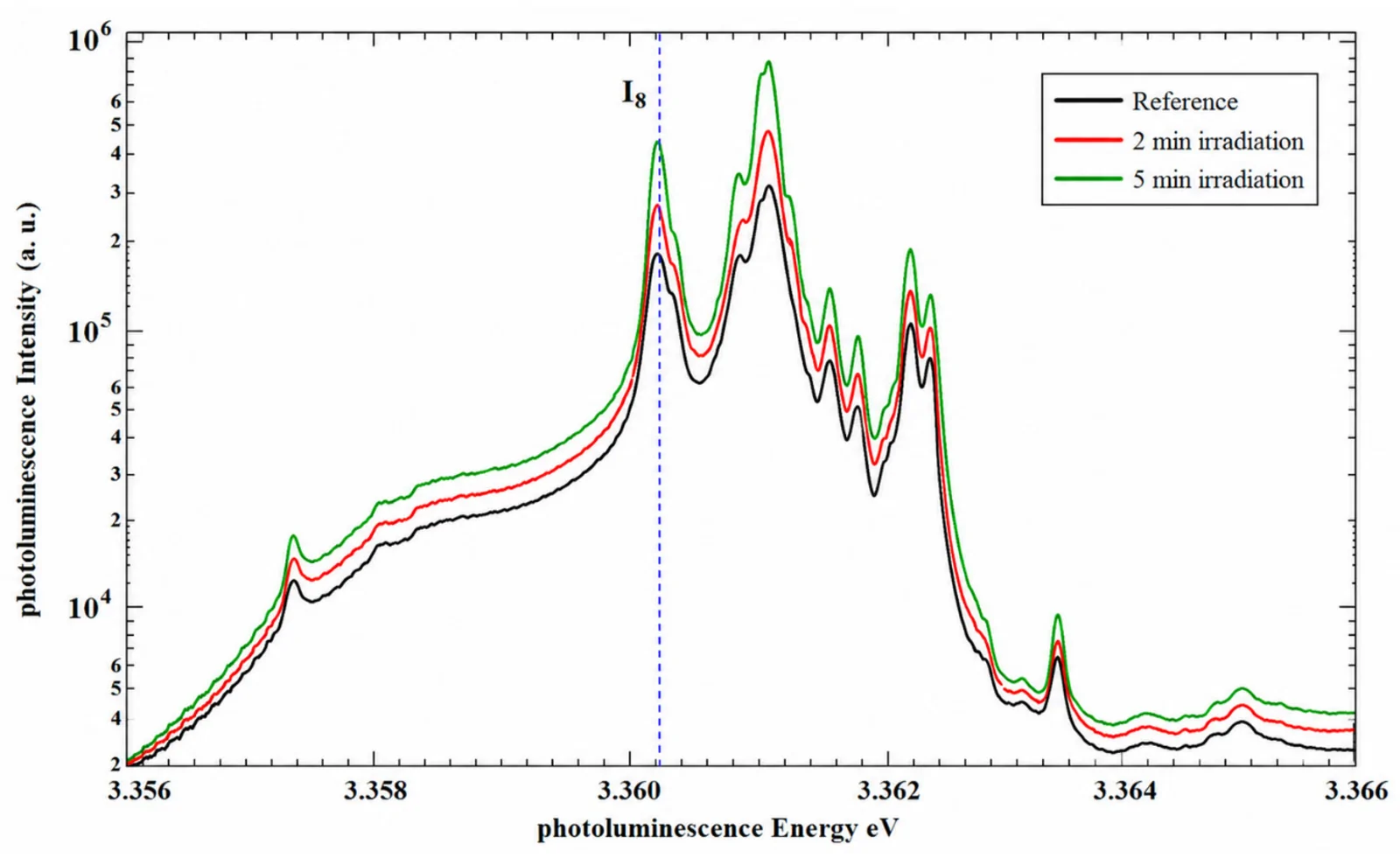
Low-temperature photoluminescence spectra of the unirradiated reference sample and Zn-polar ZnO samples following 2 and 5 min of fast-neutron irradiation. The spectra were acquired under identical experimental conditions and are presented on the same intensity scale to enable direct comparison of the Ga-related donor-bound exciton (I_8_). A progressive enhancement in the I_8_ emission intensity is observed with increasing irradiation duration, while the overall spectral profile remains essentially unchanged.

Figure 2 compares the low-temperature photoluminescence spectra of the unirradiated reference sample and Zn-polar samples following 2 and 5 min of fast-neutron irradiation under identical experimental conditions. The three spectra exhibit nearly identical spectral profiles, indicating that fast-neutron irradiation did not introduce new radiative transitions or significantly alter the overall near-band-edge emission characteristics of the ZnO crystal. Instead, the principal experimental observation is a systematic increase in the intensity of the Ga-related donor-bound exciton (I_8_), with the enhancement becoming progressively greater as the irradiation duration increased from 2 to 5 min.

In addition to the increased I_8_ emission intensity, no measurable shift in the emission energy or noticeable broadening of the excitonic features was observed. The preservation of the spectral shape indicates that the irradiation primarily influenced the intensity of the donor-bound excitonic emission rather than fundamentally modifying the optical structure of the near-band-edge photoluminescence.

### 3.2. Quantitative Enhancement of the I_8_ Donor-Bound Exciton

To quantify the influence of irradiation duration on the optical response of ZnO, the photoluminescence spectra were compared under identical experimental conditions using the peak intensity of the Ga-related donor-bound exciton (I_8_). Relative to the unirradiated reference sample, the I_8_ emission intensity increased by approximately 33% following 2 min of fast-neutron irradiation and by approximately 82% following 5 min of irradiation.

The progressive increase in I_8_ intensity demonstrates a clear dependence of the photoluminescence response on irradiation duration. Importantly, the enhancement occurred without measurable changes in the emission energy or overall spectral profile, indicating that the dominant effect of fast-neutron irradiation under the present experimental conditions was an increase in the intensity of the Ga-related donor-bound exciton rather than the formation of new luminescence bands or significant modification of the near-band-edge emission structure.

The photoluminescence measurements therefore demonstrate a reproducible, irradiation-time-dependent enhancement of the Ga-related I_8_ donor-bound exciton in hydrothermally grown ZnO. These experimental observations form the basis for the mechanistic interpretation presented in the Section 4.

## 4. Discussion

The photoluminescence results presented in this study demonstrate that fast-neutron irradiation produces a reproducible and irradiation-time-dependent enhancement of the Ga-related donor-bound exciton (I_8_) in hydrothermally grown ZnO bulk single crystals. Relative to the unirradiated reference sample, the I_8_ emission intensity increased by approximately 33% after 2 min of irradiation and by approximately 82% after 5 min, while the overall near-band-edge spectral profile remained essentially unchanged. The absence of significant peak shifts, spectral broadening, or additional emission bands indicates that fast-neutron irradiation primarily modifies the intensity of the donor-bound excitonic emission rather than fundamentally altering the optical characteristics of the ZnO crystal.

These observations are consistent with increased Ga-related donor activity following irradiation and provide evidence that fast neutrons can efficiently modify the low-temperature optical response of ZnO within relatively short exposure times. Compared with our previous investigation of slow-neutron irradiation, which required irradiation periods of several days to achieve measurable enhancement of the I_8_ emission, the present results demonstrate that comparable optical modifications can be produced within only a few minutes using high-energy fast neutrons. However, because the two studies employed different neutron energies, fluences, and irradiation conditions, this comparison should be interpreted qualitatively rather than as a direct measure of irradiation efficiency.

The enhanced I_8_ donor-bound exciton observed following fast-neutron irradiation is consistent with increased Ga-related donor activity within the ZnO lattice. A plausible explanation is neutron-induced transmutation of zinc isotopes into gallium donor species, a mechanism previously proposed for neutron-irradiated ZnO. Gallium is a well-established shallow donor in ZnO, and the I_8_ exciton has been widely attributed to gallium donor-bound excitonic recombination. Consequently, an increase in optically active gallium donors would be expected to enhance the intensity of the I_8_ emission.

In addition to neutron transmutation, fast neutrons possess sufficient energy to produce atomic displacements, lattice distortions, and point defects throughout the crystal volume. These irradiation-induced defects may influence carrier localization, modify local recombination pathways, or alter the population of radiative recombination centers, thereby contributing to the observed increase in excitonic emission intensity. The preservation of the overall spectral profile following irradiation suggests that these structural modifications do not fundamentally alter the near-band-edge luminescence but instead influence the efficiency of donor-bound excitonic recombination.

Although the present photoluminescence measurements are consistent with increased Ga-related donor activity, they do not constitute direct evidence of gallium formation. Confirmation of neutron-induced transmutation would require complementary compositional characterization using techniques such as secondary ion mass spectrometry (SIMS), inductively coupled plasma mass spectrometry (ICP-MS), or atom probe tomography. Consequently, the proposed Zn-to-Ga transmutation mechanism should be regarded as the most plausible interpretation of the present optical observations rather than definitive proof.

The proposed transmutation mechanism is directly related to the method used to generate the fast-neutron beam. In the present study, fast neutrons were produced at the University of Washington cyclotron through deuteron-induced reactions on a beryllium target. The primary neutron-producing reaction can be represented as:

H12+Be49→→B510+n + 014.36 MeV

A deuteron consists of a bound proton and neutron and can undergo breakup interactions when incident on a target nucleus, producing energetic neutrons over a broad energy spectrum. These breakup reactions contribute significantly to the fast-neutron flux employed in the present work and can be represented by:

XZA+H12→→n01+YZ+1A+1

XZA+H12→→p11+YZA+1

The resulting neutron beam, with an average energy of approximately 22 MeV, provides the energetic particles responsible for the neutron–matter interactions discussed in the following section.

The enhanced I_8_ emission observed following irradiation is consistent with the well-established neutron transmutation reactions of naturally occurring zinc isotopes. As summarized in Table 1, neutron capture by the ^68^Zn and ^70^Zn isotopes produces the radioactive intermediates ^69^Zn and ^71^Zn, which subsequently undergo β-decay to form the stable gallium isotopes ^69^Ga and ^71^Ga. Because gallium is a shallow donor in ZnO, these transmutation reactions provide a physically plausible explanation for the increased Ga-related donor activity inferred from the photoluminescence measurements.

The present work extends our previous slow-neutron investigation by demonstrating that similar transmutation-related optical behavior can be achieved using high-energy fast neutrons over irradiation periods of only a few minutes. While the photoluminescence data support this interpretation, direct confirmation of irradiation-induced gallium formation would require complementary compositional techniques such as SIMS or ICP-MS [42,43,44,45,46].

**Figure 3:**
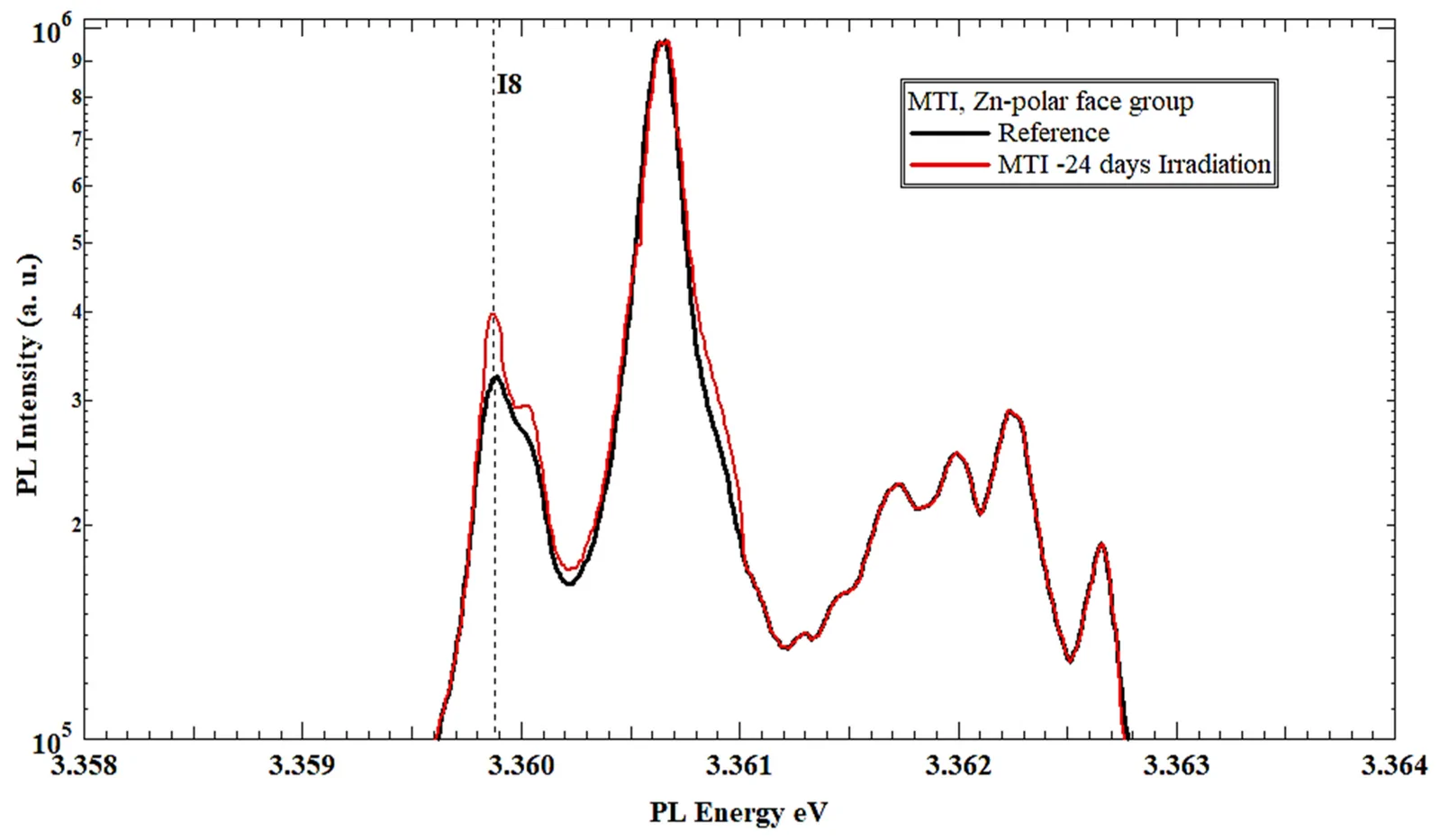
Low-temperature (4 K) photoluminescence spectra of hydrothermally grown ZnO bulk single crystals measured from the Zn-polar (0001) face before and after prolonged slow neutron irradiation for up to 24 days, reproduced from Ref. [10]. Gradual enhancement of the gallium-related I8 excitonic emission is observed with increasing irradiation duration.

The influence of neutron energy on the optical response of ZnO is illustrated by comparing the present fast-neutron results with our previously reported slow-neutron irradiation study, as shown in Figure 3. Although the irradiation conditions differ substantially in terms of neutron energy, fluence, and exposure duration, both studies exhibit the same qualitative behavior: an enhancement of the Ga-related donor-bound exciton (I_8_). This consistency supports the interpretation that neutron irradiation promotes increased Ga-related donor activity within the ZnO lattice.

A notable distinction between the two investigations is the irradiation time required to produce measurable optical enhancement. In the previous slow-neutron study, enhancement developed progressively over several days, whereas the present work demonstrates significant enhancement after only 2 and 5 min of fast-neutron exposure. This difference is likely associated with the substantially higher neutron energy employed in the present study, which increases the probability of neutron–lattice interactions capable of producing atomic displacements, defect formation, and transmutation-related processes. Nevertheless, because the neutron spectra, fluences, and irradiation geometries differ between the two experiments, the comparison should be regarded as qualitative rather than quantitative.

**Figure 4:**
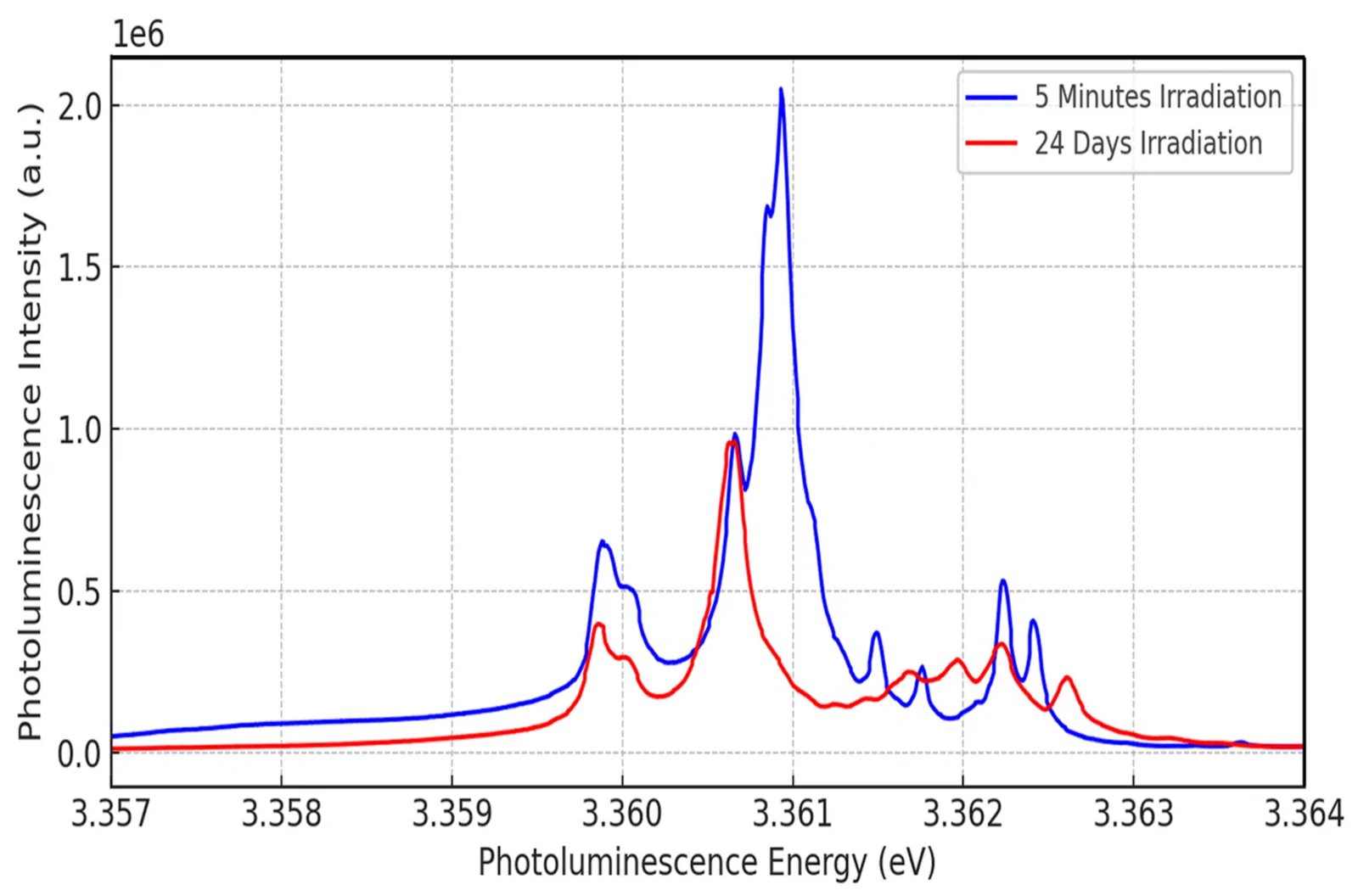
Comparison of low-temperature photoluminescence spectra obtained from Zn-polar (0001) ZnO bulk single crystals following 5 min of fast neutron irradiation and 24 days of slow neutron irradiation [10]. Fast neutrons produce substantially greater enhancement of the gallium-related I_8_ excitonic emission within a significantly shorter irradiation duration.

Compared with charged-particle irradiation, fast neutrons possess substantially greater penetration depths and are therefore capable of interacting throughout a much larger volume of the crystal. Whereas energetic ion beams primarily modify near-surface regions through collision cascades and atomic mixing, fast neutrons can induce lattice modifications within the bulk material. Consequently, fast-neutron irradiation represents a complementary approach for modifying semiconductor materials, particularly when bulk optical or electronic properties are of interest. The present results demonstrate that this approach is capable of producing significant enhancement of the Ga-related donor-bound exciton in hydrothermally grown ZnO following only short irradiation periods as illustrated in Figure 4 [42,43,44,45,46].

The present study demonstrates that fast-neutron irradiation provides an efficient route for modifying the low-temperature optical response of hydrothermally grown ZnO bulk single crystals. The reproducible enhancement of the Ga-related I_8_ donor-bound exciton, together with the preservation of the overall near-band-edge spectral characteristics, indicates that fast neutrons can selectively influence donor-related radiative recombination without substantially degrading the optical quality of the material. Although the photoluminescence measurements are consistent with neutron-induced transmutation and irradiation-assisted defect evolution, direct verification of these processes requires complementary structural and compositional characterization. Future investigations combining photoluminescence spectroscopy with techniques such as SIMS, XRD, Raman spectroscopy, transmission electron microscopy, and atom probe tomography will provide a more comprehensive understanding of the mechanisms governing neutron-induced modification of ZnO. Such studies may further establish fast-neutron irradiation as a valuable tool for controlled defect and donor engineering in wide-bandgap semiconductor materials.

## 5. Conclusions

Fast-neutron irradiation has been shown to produce a reproducible enhancement of the Ga-related donor-bound exciton (I_8_) in hydrothermally grown ZnO bulk single crystals following irradiation periods of only 2 and 5 min. Low-temperature photoluminescence measurements revealed progressive increases in the I_8_ emission intensity of approximately 33% and 82%, respectively, while the overall near-band-edge spectral profile remained essentially unchanged. These observations indicate that fast-neutron irradiation can modify donor-related optical activity without significantly altering the fundamental excitonic emission characteristics of the ZnO crystal.

The enhanced I_8_ emission is consistent with increased Ga-related donor activity arising from neutron-induced zinc-to-gallium transmutation together with irradiation-induced lattice modifications that may promote donor-bound excitonic recombination. Although direct confirmation of these mechanisms requires complementary structural and compositional characterization, the present photoluminescence results demonstrate that fast neutrons provide an efficient means of modifying the optical response of ZnO over remarkably short irradiation durations.

Compared with our previous slow-neutron investigation, the present study demonstrates that significant enhancement of the I_8_ exciton can be achieved using high-energy fast neutrons within only a few minutes of exposure. While the two irradiation conditions are not directly comparable because of differences in neutron energy, fluence, and experimental geometry, the results highlight the considerable potential of fast-neutron irradiation for rapidly modifying the optical behavior of ZnO.

Overall, this work provides new insight into the interaction of fast neutrons with wide-bandgap oxide semiconductors and establishes a foundation for future investigations combining optical spectroscopy with structural and compositional characterization to further elucidate the mechanisms governing neutron-induced donor activation and defect engineering.

## Figures and Tables

**Table 1:** Nuclear reactions of Zn isotopes with neutrons and the transmute atom [10].

Isotopes	1st and 2nd Nuclear Reactions	Half-Life (T_1/2_)
^64^Zn	Zn3064+n1→Zn3065+γ Zn3065+e−1→Cu2965+γ	244.26 days
^66^Zn	Zn3066+n1→Zn3067+γ Zn3067 Stable	-
^67^Zn	Zn3067+n1→Zn3068+γ Zn3068 Stable	-
^68^Zn	Zn3068+n1→Zn3069+γ Zn3069→Ga3169+β−1	56.4 min
^70^Zn	Zn3070+n1→Zn3071+γ Zn3071→Ga3171+β−1	2.45 min

## Data Availability

The data of this research is available upon request.
